# Estimation of woody plant species diversity during a dry season in a savanna environment using the spectral and textural information derived from WorldView-2 imagery

**DOI:** 10.1371/journal.pone.0234158

**Published:** 2020-06-08

**Authors:** Emmanuel Fundisi, Walter Musakwa, Fethi B. Ahmed, Solomon G. Tesfamichael

**Affiliations:** 1 Department of Geography, Environmental Management and Energy Studies, University of Johannesburg, Johannesburg, South Africa; 2 Department of Urban and Regional Planning, Future Earth and Ecosystems Services Research Group, Doornfontein Campus, University of Johannesburg, Johannesburg, Gauteng, South Africa; 3 School of Geography, Archaeology and Environmental Studies, University of Witwatersrand, Johannesburg, South Africa; Aarhus University, DENMARK

## Abstract

Remote sensing techniques are useful in the monitoring of woody plant species diversity in different environments including in savanna vegetation types. However, the performance of satellite imagery in assessing woody plant species diversity in dry seasons has been understudied. This study aimed to assess the performance of multiple Gray Level Co-occurrence Matrices (GLCM) derived from individual bands of WorldView-2 satellite imagery to quantify woody plant species diversity in a savanna environment during the dry season. Woody plant species were counted in 220 plots (20 m radius) and subsequently converted to a continuous scale of the Shannon species diversity index. The index regressed against the GLCMs using the all-possible-subsets regression approach that builds competing models to choose from. Entropy GLCM yielded the best overall accuracy (adjusted R^2^: 0.41−0.46; Root Mean Square Error (RMSE): 0.60−0.58) in estimating species diversity. The effect of the number of predicting bands on species diversity estimation was also explored. Accuracy generally increased when three–five bands were used in models but stabilised or gradually decreased as more than five bands were used. Despite the peak accuracies achieved with three–five bands, performances still fared well for models that used fewer bands, showing the relevance of few bands for species diversity estimation. We also assessed the effect of GLCM window size (3×3, 5×5 and 7×7) on species diversity estimation and generally found inconsistent conclusions. These findings demonstrate the capability of GLCMs combined with high spatial resolution imagery in estimating woody plants species diversity in a savanna environment during the dry period. It is important to test the performance of species diversity estimation of similar environmental set-ups using widely available moderate-resolution imagery.

## Introduction

Natural environments provide vital ecosystem services such as primary energy, habitat to fauna, maintaining of hydrological cycles, protection of biological diversity, medicinal benefits, recreation, aesthetic values, etc [1–4]. Maintaining the heterogeneity and complexity of vegetation composition in these environments is therefore critical to ensure the sustained functioning of such ecosystems [3]. Unfortunately, anthropogenic activities and natural processes are threatening biodiversity and associated ecosystem services, particularly in areas close to human settlements [4–7]. An essential first step in the management of biodiversity is through accounting and monitoring of vegetation composition existing in a given area of interest. In Africa, inventories on plant species diversity are often out-of-date and/or unavailable [8]. Field inventory for characterising vegetation composition is expensive, labour intensive and time-consuming, and thus is inefficient in complex heterogeneous ecosystems [3,5,9–12].

Remote sensing overcomes the difficulties of field-based inventory and has become a primary tool in species diversity estimation and ecosystem structure assessments [9–11]. In particular, optical remote sensing utilises the sensitivity of spectra to biochemical and structural characteristics to distinguish vegetation types [12]. Advances in spectral and spatial resolution of remote sensing have allowed for more efficient species diversity estimation in different environments including grasslands (e.g., [7,13], temperate forest (e.g., [14–16], wetlands (e.g., [17,18], tropical forest (e.g., [19–21] and savanna (e.g., [22–24].

The traditional remote sensing-based species classification approaches identify a defined number of classes, irrespective of the spatial resolution of remotely-sensed data [9,21,22,25]. Such approaches, therefore, can underestimate the number of species that potentially exist in a given environment. Statistical modelling approaches such as the Shannon diversity index offer an alternative approach by converting categorical species data into continuous diversity scale [25–27], thus eliminating the restriction on the number of species that can be estimated for a given area [13,17,28–30]. A number of studies have applied continuous-scale metrics derived from species count data to quantifying woody plant species diversity in the savanna vegetation type [24,27,31–35].

Vegetation indices (e.g., Normalised Vegetation Index, NDVI) have been used as the common source of data to quantify vegetation species diversity on a continuous scale [24,28,36,37]. It should be noted that Vegetation Indices (VIs) primarily capture vegetation vigour or amount rather than species diversity [9–11]. Thus, a plot with high species diversity but low vigour (low index value) can be misinterpreted as having low species diversity while a plot containing a single or few plants with high vigour can be misinterpreted as being species-rich [10,38]. It is therefore important to test the performance of individual spectral bands rather than relying on VIs, to quantify species diversity.

A further challenge associated with reliance on vegetation indices is that the indices logically work well in high-photosynthesis-activity periods of the year [38–40]. For instance, [15,24,41] compared species diversity estimation in the dry versus wet season and found better accuracies in the wet season. There is, therefore, a need to exploit the information offered by individual bands of satellite imagery to assess species diversity in the dry season within the savanna environment. While biodiversity assessment is ideal in the wet (vigorous seasons), placing focus on the dry season has operational and ecological benefits. Operationally, remotely-sensed data acquired during dry seasons are less affected by clouds and therefore provide readily interpretable information [38]. Ecologically, dry season biodiversity assessment is vital as part of continuous monitoring strategies such as phenological tracking [6,22] and land degradation facing the savanna ecosystem [23,42].

This study, therefore, aims to assess the performance of individual bands of WorldView-2 imagery in quantifying woody plant species diversity in a dry season within the savanna region in South Africa. The study uses various GLCMs of the WorldView-2 bands as predictors to estimate field-derived species diversity expressed in the Shannon diversity index. Our study builds on [33] who extracted all eight GLCMs but only used one band (near-infrared) of a Landsat image from the wet season of an American savanna to estimate plant species diversity. Our study also differs from [43] that used fewer GLCMs and coarser spatial resolution (Landsat data). We apply the all-possible-subset regression approach to exploit the large number of models that can be created using individual bands as predictors. This specific regression approach provides alternative competing models from which a reasonable model can be selected, therefore it was selected for this study. Satisfactory findings in this study have important implications for conservation efforts that require vegetation species monitoring, irrespective of the season [44].

## Materials and methods

### Study area

**Fig 1:**
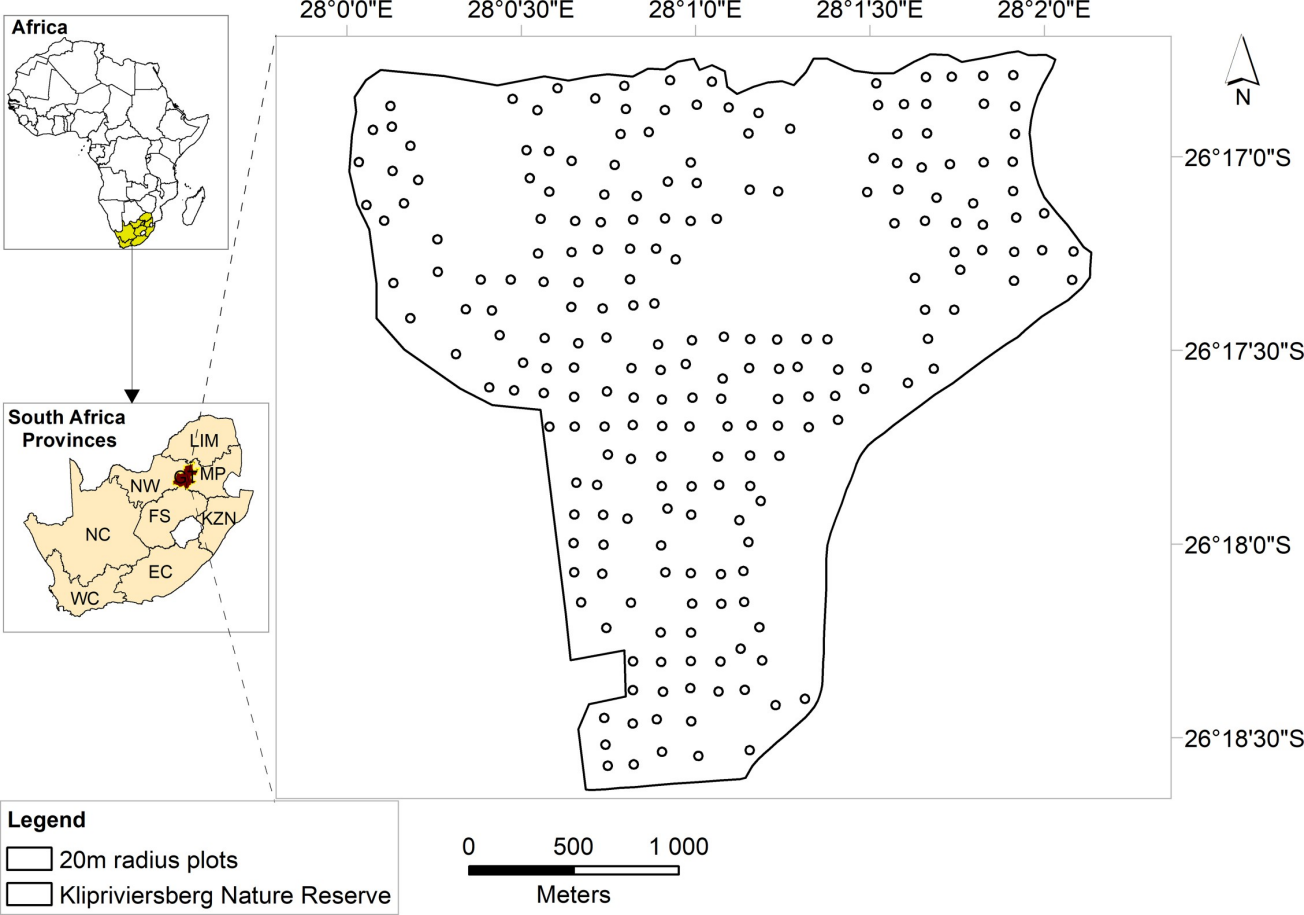
Klipriviersberg Nature Reserve and the distribution of sampling plots used in the study.

The Klipriviersberg Nature Reserve (KNR), south of Johannesburg, South Africa, was used for the study. The reserve was proclaimed for conservation purposes in 1984 and covers approximately 651 hectares (Fig 1). KNR, administered by the City Parks of the City of Johannesburg, is a public park open to visitors at no cost. Different activities such as hiking and research work are allowed in the reserve without the need or written permission from the management provided that the regulations governing the reserves’ natural resources are observed at all times. Vegetation types in the reserve include Andesite Mountain Bushveld and Clay Grassland which are associated with a savanna environment [45]. The altitude of the area ranges between 1540 m in the south and 1790 m in the north, with a mean annual rainfall of 624–802 mm [46]. The study area experiences warm to hot summers and cold nights in winter, with a mean annual temperature ranging between 17°C and 26°C in summer and 5°C and 7°C in winter [47]. The geology types found in the area, which lead to the floristic structure of the reserve, include quartzites, conglomerates and dolomites [48].

### Field data

**Fig 2:**
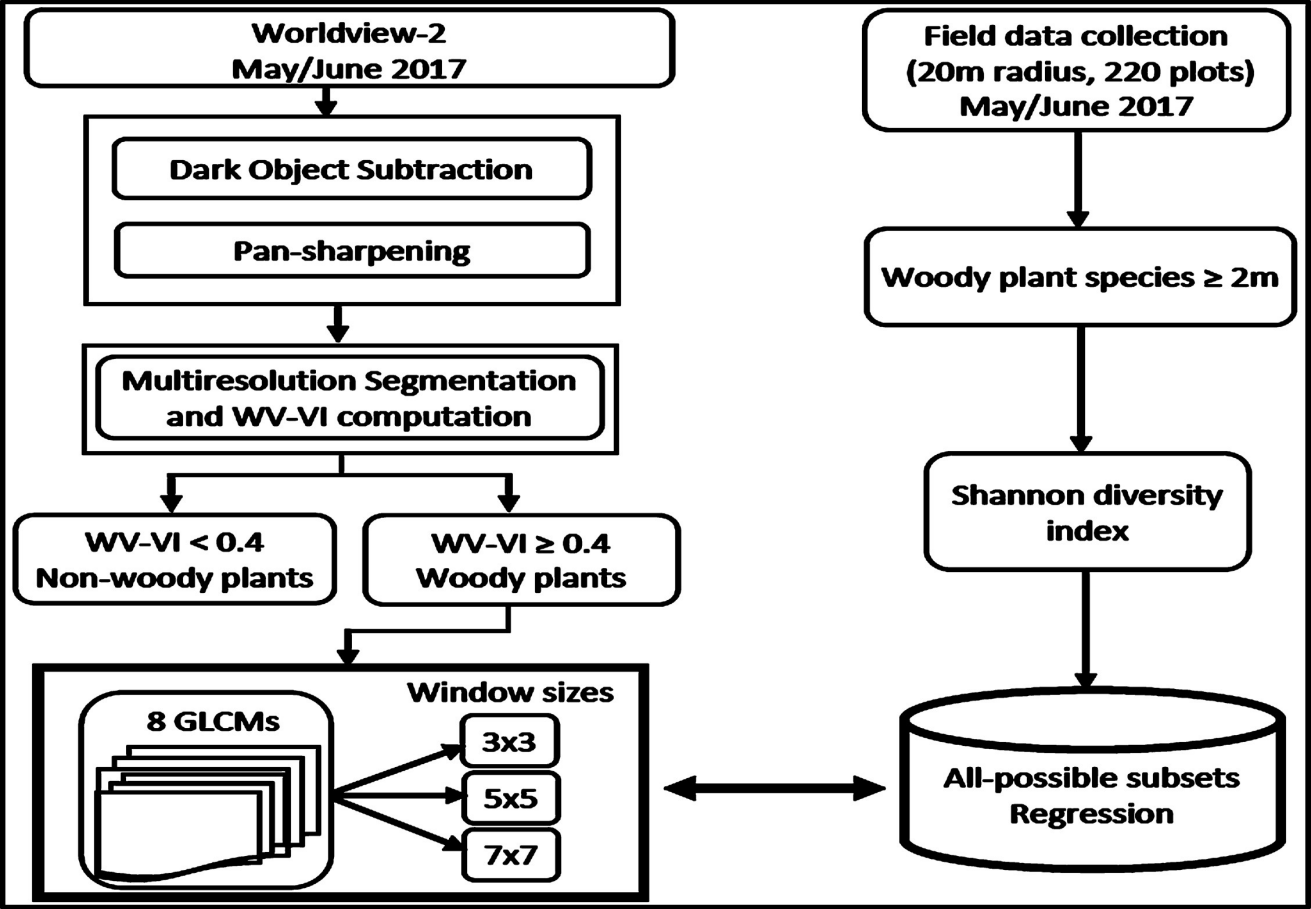
Flow chart summarizing the methodology used in the study area.

Fig 2 provides a summary of the methodology followed in the study to estimate woody plant species diversity. Initially, a grid of 240 points distributed at approximately 170 m intervals in the north-south and east-west directions were generated using the fishnet tool in ArcGIS (ESRI® ArcGIS 10.6, Redlands, California, USA). The point coverage was exported into a Global Position System (GPS) (Garmin, GPSMAP® 64, Kansas, USA) and located in the field. Points which did not have woody plant species in their vicinity were removed from the enumeration resulting in 220 number of points available for the survey. Field surveys were done between May and June 2017, representing the dry winter season in the area [47]. A buffer with a 20 m radius was created around each point; this size was specified to accommodate not only multiple pixels of WorldView-2 imagery, but also coarser-resolution remotely sensed data that will be used for further study. The plot size was also deemed large enough to contain as many woody plant species as possible.

Unique woody plant species ≥ 2 m height were recorded in each plot (Fig 2) guided by the KNR field surveyors and the reserve’s species inventory database. The total number of distinct species in all plots was 26, while the plot-level minimum and maximum count of distinct woody plant species were 1 and 9, respectively. In addition to species uniqueness, the survey recorded the total number of woody plants ≥ 2 m in each plot. The species diversity was then converted to a continuous scale by applying the Shannon diversity index [49] for use in regression modelling. The Shannon index (Eq 1) is advantageous in that it incorporates the relative abundance and evenness of species, and therefore does not favour common or rare species [33,50]. The minimum, maximum, standard deviation of the Shannon diversity index in the sampled plots were 0.15, 2.86 and 0.78, respectively.

$$Shannon\;diversity\;index=-\sum_{i=1}^{s}pln\;(p_{i})$$ (1)

where *p* is the number of plants of a species divided by the total number of all plants, ln is the natural log, s is the number of species.

### WorldView-2 image

WorldView-2 imagery covering the visible to near-infrared range of the electromagnetic spectrum was obtained for the same dry winter period (May−June 2017) as the field survey period. WorldView-2 image has high spectral and spatial resolutions making it effective for fine-scale woody plant species diversity estimation [51]. Specifically, the image has eight multispectral bands between 0.40 and 1.04 μm at 1.8 m spatial resolution and a panchromatic band covering 0.45−0.80 μm at 0.5 m spatial resolution (DigitalGlobe, www.digitalglobe.com). The bands vary in width with the yellow (0.59 and 0.63 μm) and red edge (0.71 and 0.75 μm) bands being narrower than the others. The coastal band (0.40−0.46 μm) was excluded from the analysis due to its relative sensitivity to atmospheric interferences [50]. Following a comparison of Dark Object Subtraction (DOS), [52], Fast Line-of-sight Atmospheric Analysis of Hypercubes (FLASH), [53] and QUick Atmospheric Correction (QUAC), [54] methods that yielded similar reflectance values (Pearson’s correlation, *r* = 0.99)). We applied the DOS method implemented in ENVI 5.3 ©2015 (Exelis Visual Information Solution Inc., Boulder, Colorado). Subsequently, the spatial resolution of the atmospheric corrected multispectral bands was pan-sharpened to 0.5 m. (Fig 2). The reliability of our pan-sharpening was ascertained by comparing the results with the already pan-sharpened image provided by the data supplier (DigitalGlobe, www.digitalglobe.com).

### Woody vegetation extraction from WorldView-2 image

Pixels representative of woody vegetation plants were extracted prior to derivation of GLCMs that were used in the regression analysis. Previous studies have shown the effectiveness of NDVI thresholding to separate woody and non-woody plants (e.g., [55–57]. The approach assumes that woody plants including trees, bushes and shrubs with relatively dense foliage have greater NDVI than non-woody plants [57–60]. In this study, we computed WorldView-2 Improved Vegetation Index (WV-VI) [61] that combines the near-infrared 2 and the red bands in a similar formula as the NDVI. The narrowly focused near-infrared 2 band (0.86−1.04 μm) used in WV-VI has a higher reflectance value than the traditional broad near-infrared band range used in the NDVI [62].

Parallel to WV-VI derivation, objects were created from the multispectral image of WorldView-2 using multiresolution segmentation, following the previous studies that showed good woody plants identification capability using object-based classification [63–66]. The segmentation was applied in eCognition Developer® 9.01 (Trimble Germany GmbH, Arnulfstrasse 126, 80636 Munich, Germany). The WV-VI values were subsequently averaged per segment [67]. After iterative and independent trials, segments with WV-VI ≥ 0.4 were determined as woody plants separating them from non-woody plants (Fig 2). Segments of woody plants and non-woody features were confirmed by consulting with plot observations that were taken during field surveys.

### Gray Level Co-occurrence Matrix

GLCMs were quantified from individual bands of WorldView-2 imagery within woody segments. GLCM is a statistical method used to examine the texture of pixels within a specific neighbourhood [68]. Since the statistic quantifies how often unique combinations of pixel brightness values (gray levels) occur in an image, it signifies underlying physical variations in the image and thus reveals the structural arrangements of the surface and their relationship to the surrounding environment [69]. Typically, GLCM implements a matrix scenario and it is computed by considering four different directions (0°, 45°, 90°, and 135°) between neighbouring cells that are separated by a certain distance [68,69]. This study extracted eight GLCM statistics derived from each WorldView-2 band using ENVI 5.3 ©2015 (Exelis Visual Information Solutions. Inc Boulder, Colorado).

The GLCMs were quantified in three kernel sizes: 3×3, 5×5 and 7×7. These window sizes were specified considering the spatial resolution of the image (0.5 m) and the varying canopy sizes of woody vegetation observed during the field survey. In addition, plants were observed as isolated individuals or in patches–as is common in savanna environments [28,32,33]. It is unclear how such arrangements influence the detection capability of different window sizes. It is important to ascertain that these kernel sizes exhibit variation and thereby may warrant different species diversity estimations. A simple Pearson’s correlation analysis of GLCMs among the different kernel sizes using Entropy and three selected bands (Green, Yellow and Near-infrared) resulted in *r* ≤ 0.5 for a number of comparisons, indicating the difference between the window sizes.

### Statistical analysis

This study used an all-possible-subsets regression modelling approach to estimate the Shannon diversity index derived from the field data using GLCM values (Table 1) derived from WorldView-2 bands at different window sizes (3×3, 5×5 and 7×7) as predictors. All-possible-subsets regression tests all possible combinations of explanatory variables to develop estimation models from which favourable models can be selected [70]. A key advantage of the approach is that it does not require significance-testing that can be influenced by certain values in the samples that may or may not be representative of the population [70]. Instead, the approach provides all alternative models from which decisions can be made based on knowledge of the population from which the samples were drawn [70]. The all-possible-subset regression approach builds 2^*n*^ - 1 models from *n* number of explanatory variables [71]. In this study, the alternative models for estimating the Shannon diversity index were grouped and compared per GLCM window size. That is, the seven bands (explanatory variables) within the 3×3 window had 127 competing models; a similar principle applied to 5×5 and 7×7 windows respectively.

**Table 1:** A list of GLCM texture features extracted from eight WorldView-2 bands and used in this analysis derived from [69].

GLCM statistics and formula	Description
$Entropy=\sum_{i,j=0}^{N_{g}-1}g^{2}(i,j)In\;g(i,j)$	Entropy measures the occurrence of random pair of pixels
$Second\;Moment=\sum_{i,j=0}^{N_{g}-1}g^{2}(i,j)$	Second moment measures the occurrence of a common pair of pixels
$Contrast=\sum_{i,j=0}^{N_{g}-1}{\left(i,j\right)}^{2}g(i,j)$	Contrast measures change in gray level between adjoining pixels and the weighting on pixel pairs increases exponentially.
$Correlation=\sum_{i,j=0}^{N_{g}-1}(i-\mu )(j-\mu )g(i,j)/\sigma^{2}$	Correlation measures the linear dependency of a pair of pixels in the image.
$Variance=\sum_{i,j=0}^{N_{g}-1}{\left(i=\mu \right)}^{2}g(i,j)$	Variance measures dispersion of gray level values around the mean
$Homogeneity=\sum_{i,j=0}^{N_{g}-1}\frac{1}{1+{\left(i-j\right)}^{2}}g(i,j)$	Homogeneity measures image pixel similarity and it is sensitive to the presence of near diagonal elements in a GLCM
$Mean=\sum_{i=1}^{N_{g}}\sum_{i=1}^{N_{g}}i*P(i,j)$	Mean measures the average GLCM of gray level values in an image
$Dissimilarity=\sum_{i=1}^{N_{g}}\sum_{i=1}^{N_{g}}P(i,j)|i-j|$	Dissimilarity measures the amount of change in nearby pixels with the weighting on pixel pairs increasing linearly

where *N*_*g*_ is the number of gray levels, *g*(*i*, *j*) is the entry (*i*, *j*) in the GLCM, μ is the GLCM mean, *σ*^2^ is the GLCM variance and *P* is the proportion of occupancy of each pixel value.

By selecting the best model from the alternative models, in this study sought to achieve good accuracy with few predictors [70] using a combination of statistical indicators. Firstly, the Akaike Information Criterion (AIC) that measures the distance between a model and an ideal but unobservable model that created the data was used to rank the models [72]. Secondly, models were compared using adjR^2^ and RMSE values, which respectively provide an absolute measure of the explanatory power of predictors and accuracy of a model. [5,73]. Thirdly, we compared the errors of models with the smallest AIC values and explanatory variables per GLCM and window size. This approach provides more insights than the above model-fit statistics (adjR^2^ and RMSE) since it compares the direction and magnitude of errors of individual samples. In doing so, it also allows the identification of samples that do not have comparable estimates by competing models. The all-possible-subsets regression analysis was implemented using *lmSubsets* package [74] for R [75].

## Results

### Effect of window size, predictors and GLCMs on species diversity estimation

Table 2 lists the variables of the model that returned the smallest AIC value per GLCM and window size. Each selection represented the best-case AIC out of 127 competing models. The best model using entropy GLCM and a window size of 3×3 contained red, near-infrared 2, red edge and yellow bands as predictors of Shannon diversity index. Within the same GLCM group, the model developed using pixels in the 5×5 window used five bands as predictors. Looking at the rest of the GLCMs and window sizes, the best model and second-best model contained three bands as predictors. Contrast had the least number of predicting bands (one), whilst dissimilarity recorded the greatest number of predictor bands (five). The best AIC models using dissimilarity GLCM had five predictors for each window size, while the best models using mean GLCM used the same bands as predictors (green, red edge, yellow) for all window sizes. It is also important to note from Table 2 the inclusion of the yellow band as a predictor in the best-case models in five of the GLCM statistics.

**Table 2:** Predictor variables of models with the smallest AIC values for the eight GLCM statistics and three window sizes. Note that, the results presented here are the best-case scenario (smallest AIC) of 127 models per GLCM and window size.

GLCM measure	Window	Predictor bands	Smallest AIC per group of 127 competing models
	3×3	Near-infrared 2, Red, Red edge, Yellow	212.45
Entropy	5×5	Blue, Near-infrared 2, Red, Red edge, Yellow	212.13
	7×7	Near-infrared 1, Red, Yellow	213.66
	3×3	Green, Blue, Yellow	253.20
Second moment	5×5	Blue, Yellow	248.98
	7×7	Blue, Green, Yellow	250.29
	3×3	Green, Near-infrared 2, Red edge	238.08
Variance	5×5	Blue, Near-Infrared 1, Near-infrared 2	234.70
	7×7	Blue, Red edge, Green	234.59
	3×3	Near-infrared 2, Red edge, Yellow	253.12
Correlation	5×5	Blue, Yellow	254.22
	7×7	Yellow	255.66
	3×3	Blue	241.30
Contrast	5×5	Green, Near-infrared 2, Red edge	253.40
	7×7	Near-infrared 2	241.73
	3×3	Near-infrared 2, Red edge	282.64
Homogeneity	5×5	Near-infrared 2, Red edge	282.60
	7×7	Near-infrared 2, Red edge	282.43
	3×3	Blue, Near-Infrared 1, Near-infrared 2, Red edge, Yellow	235.57
Dissimilarity	5×5	Green, Near-infrared 1, Near-infrared 2, Red, Yellow	229.81
	7×7	Blue, Green, Near-infrared 1, Red edge, Yellow	226.10
	3×3	Green, Red edge, Yellow	263.47
Mean	5×5	Green, Red edge, Yellow	263.15
	7×7	Green, Red edge, Yellow	263.65

**Fig 3:**
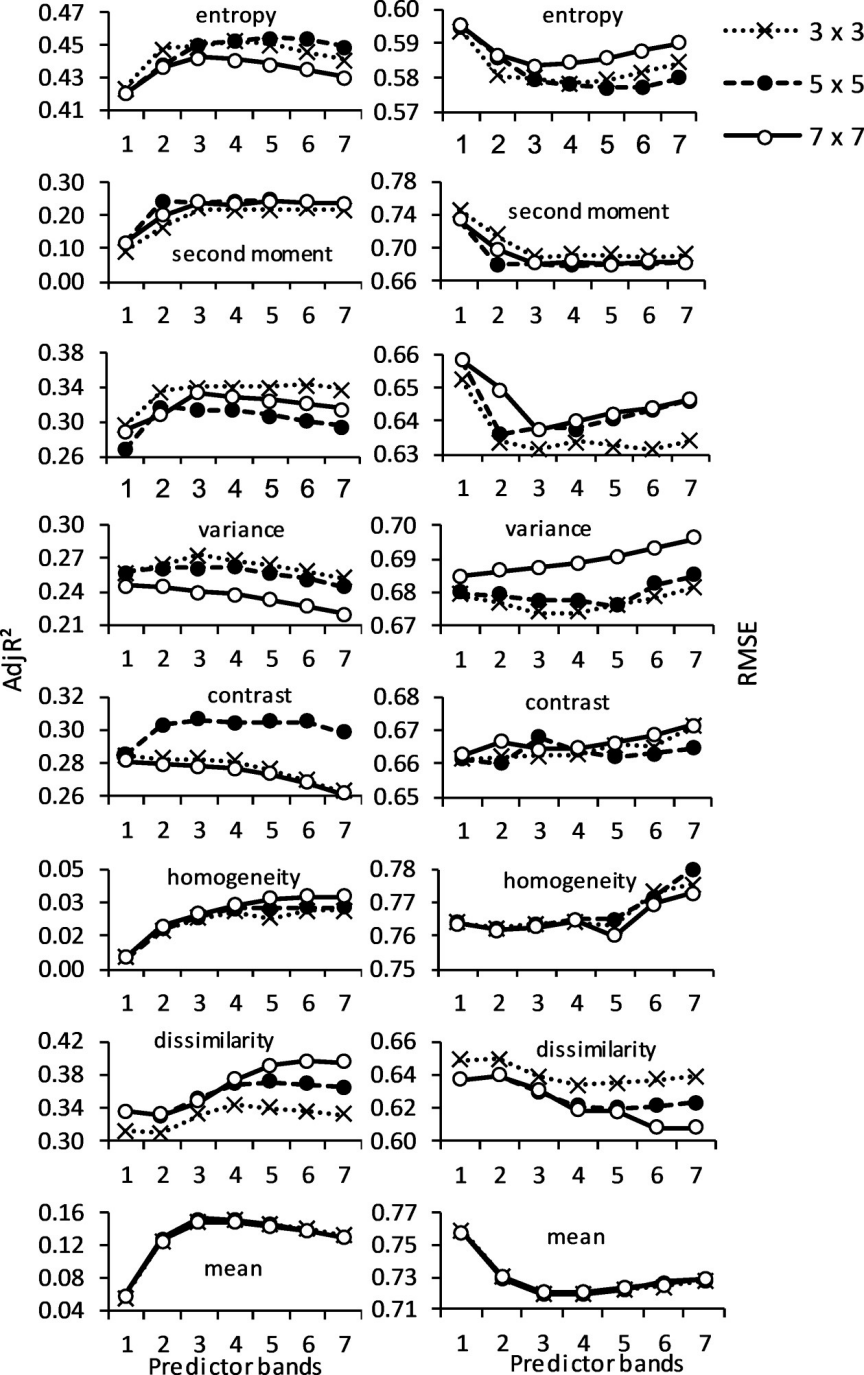
AdjR^2^ and RMSE of the best model per predictor category of GLCMs for images with 3×3, 5×5 and 7×7 window sizes.

Unfortunately, the AIC values given in Table 2 cannot be used to evaluate the relative performances of the models across GLCM and window sizes. It is, therefore, necessary to use other statistics such as adjR^2^ and RMSE to assess model accuracies and comparisons (Fig 3). Entropy GLCM achieved the best accuracies in all three windows, according to adjR^2^ (> 0.40) and RMSE (< 0.6). Specifically, entropy GLCM with a window size of 5×5 yielded the highest adjR^2^ of 0.46 and RMSE of 0.58 when five variables were used as predictors. Dissimilarity GLCM and a 7×7 window had the closest accuracy level to the entropy GLCM (adjR^2^ = 0.40; RMSE = 0.62); however, the model used six bands as predictors. Homogeneity and mean GLCMs showed the lowest prediction accuracies for all the window sizes and across the different number of predictors.

Fig 3 also shows an important trend in terms of accuracy across the number of predictors. In general, estimation accuracy increases rapidly for models that consist of three to five predictors, after which the accuracies start to level off or decrease. For instance, accuracies of models using the entropy GLCM peaked when five bands were used as predictors for the image using a 5×5 window size while the accuracies remained almost constant for all models containing six or seven predictors. The peak accuracies using the same entropy GLCM using 3×3 and 7×7 window sizes were reached when four and three predictors, were used respectively. The variance GLCM statistic that showed the third-best estimation capability after the entropy and dissimilarity statistic had models that peaked when two or three predictors were used for the three window size kernels.

**Fig 4:**
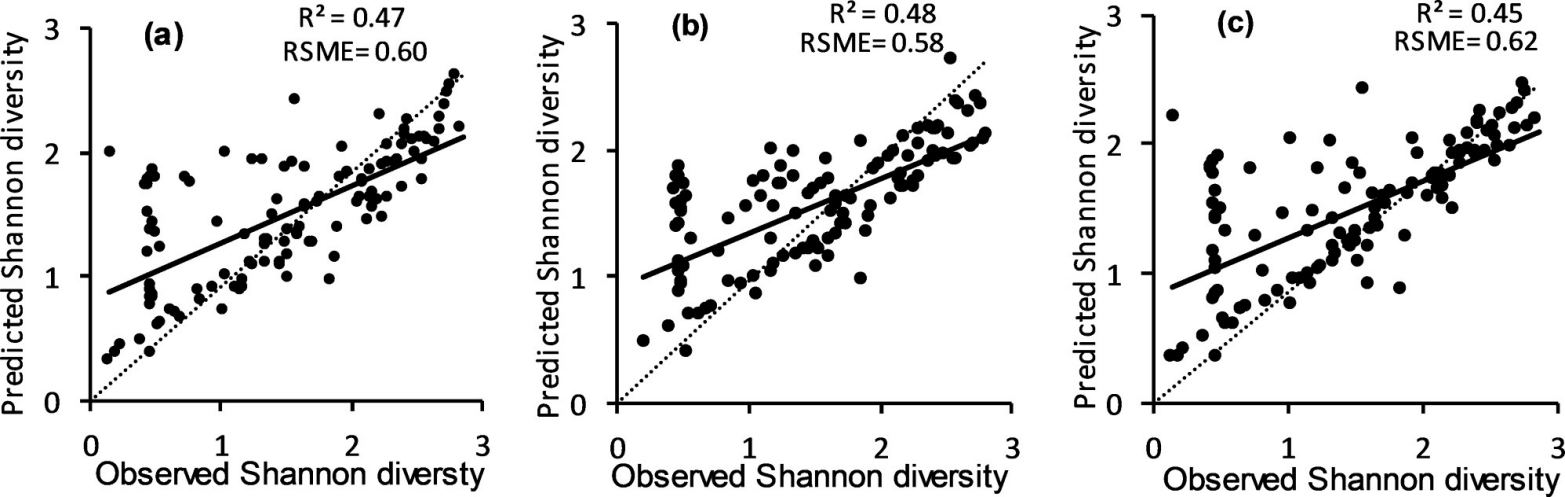
Relationship between observed and predicted Shannon species diversity index. The predicted indices were estimated using entropy GLCM derived from images using 3×3 (a), 5×5 (b) and 7×7 (c) window sizes. Note that the best estimations shown in the fig used four, five and three predictors for 3×3, 5×5 and 7×7, respectively. Dashed lines show 1:1 correspondence.

Focusing on species diversity estimation using entropy GLCM that showed the best accuracies (Fig 3), we illustrate the relationships between observed and predicted Shannon diversity index in Fig 4. Similarities can be noted among estimations using the three window sizes: 3×3 (Fig 4A), 5×5 (Fig 4B) and 7×7 (Fig 4C). One similarity is that the observed versus predicted correlations were generally comparable for the three images when compared against the ideal 1:1 correlation. The second similarity relates to the over and underestimation of the Shannon species diversity for low and high values, respectively. A closer look, however, shows the best correlation for 5×5 window size (Fig 4B). This is evidenced by the greater number of estimations deviating from the regression lines in the cases of 3×3 (Fig 4A) and 7×7 window size images (Fig 4C).

### Comparison of competing models derived from GLCMs

**Fig 5:**
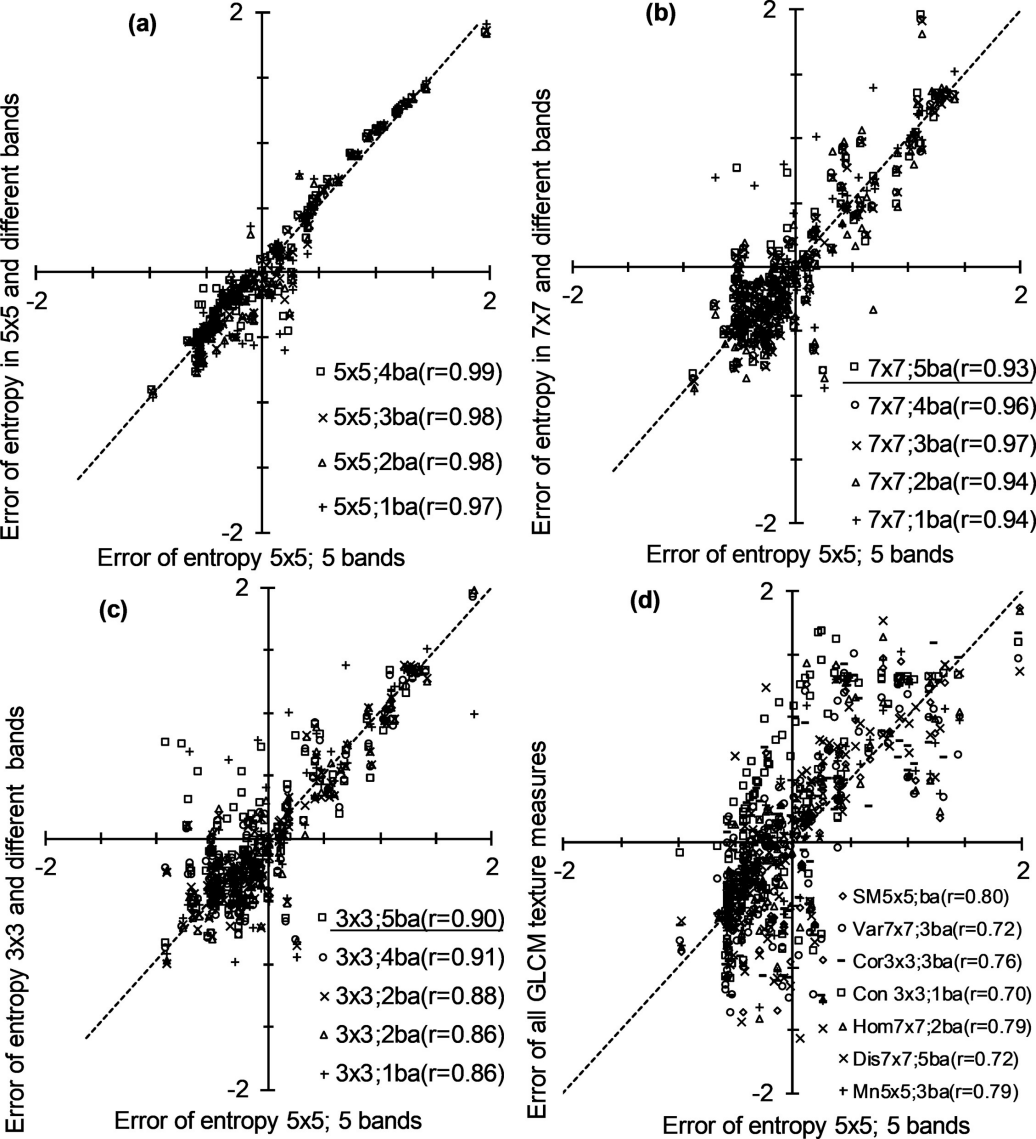
Correlation of estimation errors between selected competing models: (a) correlation between best entropy GLCM model derived from 5×5 window with 5 bands against entropy GLCM models from different number of bands within 5×5 window, (b) correlation between the best model against models derived from 7×7 window size 5 competing models, (c) correlation between best model against models derived from 3×3 window size and 5 competing models, (d) correlation between best entropy GLCM model derived from 5×5 window against seven other GLCM statistics. Underlining shows the same number of predicting bands (5) in different windows. SM = second moment, Var = variance, Cor = correlation, Con = contrast, Hom = homogeneity, Dis = dissimilarity, Mn = mean, ba = bands.

Entropy GLCM with 5×5 window size from WorldView-2 image provided better estimates of species diversity (Fig 4B compared to 4a and 4c), therefore it was used as the basis to evaluate the performances of other competing models. Three categories of comparison were made using estimation errors of all competing models as illustrated in Fig 5. The first category compared estimation errors across predictor size using the best GLCM statistic with five predictors as the reference. The results of this comparison clearly show strong similarities (r = 0.97–0.99) between the best model combining GLCM, 5×5 window size and five predicting bands with models containing fewer predictors, although the similarities show a decreasing trend as the predictor size decreases (Fig 5A).

The second category of comparison was intended mainly to assess the effect of window size on estimation residual (Fig 5B and 5C). The correlation of estimation errors of the best scenario (Entropy 5×5) was 0.93 when compared with 7×7 window size and the same predictor size (five predictor bands) while it improved when the predictors were fewer (r = 0.97 for three predictors) (Fig 5B). On the other hand, the 3×3 window size and five predictors correlated with the best scenario at r = 0.90 while the best similarity was observed when four predictors were used in the 3×3 window size (Fig 5C).

The third category of comparison was made between the overall best model (entropy GLCM using 5×5 window size image) against the best competing models from the other seven GLCM statistics as shown in Fig 5D. The correlation between entropy GLCM and the other GLCM statistics ranged between 0.70 (contrast GLCM) and 0.80 (second moment GLCM). These correlations were considerably weaker than those observed in Fig 5A–5C. This is noticeable from the error of entropy GLCM for 5×5 window size image falling mostly within the -0.65–1.5 range whilst most of the error ranged between -2 and 2 for the competing models developed from other GLCM statistics.

## Discussion

This study aimed at assessing the performance of GLCM texture values derived from individual bands of WorldView-2 imagery in quantifying woody plant species diversity in a dry season. Many studies have focused on imagery at coarser resolutions (e.g., [33,35,37,43,59,76–79]) that may ignore processes occurring at finer spatial resolutions (i.e. within pixel variation). The present study, therefore, adds to the body of knowledge by utilising finer resolution imagery and smaller filed-plots, which may lead to different observations than studies using coarser data. We converted the categorical species diversity information acquired through field survey to the continuous Shannon diversity index scale. The all-possible-subset regression approach that creates alternative estimation models was used in this study to correlate field data (expressed in Shannon index) and remotely-sensed data. [51,79–81] utilised all-possible subsets in species diversity estimation and reported its efficacy in evaluating all possible combinations of explanatory variables to select the best model. We believe that this approach is useful for exploring the suitability of the multiple sets of predictors (spectral bands, GLCM statistics and image window levels) to estimate species diversity.

### GLCMs vs. model performance

Generally, models with the best AIC ranking per window size and GLCM statistic had 3–5 bands as predictors (Table 2). Notably, the yellow band was part of the best model in most GLCM and window sizes. The ability of yellow band in discriminating plant species in dry seasons makes WorldView-2 imagery quite useful since a shortage of moisture in these seasons renders foliage yellowish [32,82]. Comparison of GLCM statistics showed entropy GLCM to be the best (Fig 4). This is not surprising given that difference in species types (mixed herbaceous−woodland plants) found in savanna creates heterogeneous environments that are capable of supporting diverse species [60].

Other better-performing GLCMs close to entropy in this study included dissimilarity, contrast and variance, all of which measure the degree of heterogeneity of the gray level and thus are capable of measuring diversity [83]. Such better performance as observed by [80,83] can be as a result of instantaneous changes in gray level values between neighbouring pixels; that portrays spatially contrasting pixel pairs. It is noteworthy to mention the weak estimation capability of some of the GLCMs, particularly homogeneity and mean (Fig 3) which do not specifically measure gray level dispersion. Homogeneity measures and represents the amount of local similarity in the image window [76] while mean measures the average gray level values in a window.

### Importance of predictor size and window size on species diversity estimation

Best estimations peaked when models contained 3–5 predictors for most GLCMs and window sizes, after which accuracies remained unchanged or decreased slightly and progressively (Fig 3). Logically, multiple individual spectral bands, should be preferable for effective identification of plant species composition [80,83–85]. This is justified by the fact that different species respond differently to incident radiant energy across the electromagnetic spectrum; hence a large number of individual spectral bands leads to a higher chance of species discrimination [9]. In connection to this, the better performance in quantifying species diversity in our study compared to [31] whose study focused on southern Africa’s savanna region (in which our study area also belongs) is attributed to the additional bands of WorldView-2 (red edge and yellow bands). These additional bands are shown to be sensitive to variations in plant condition in dry periods [28].

Despite high accuracies achieved by entropy and dissimilarity GLCM statistics, it is worth noting the higher number of predicting bands used in the latter statistic (Fig 3). The number of predictors used in a model should, therefore, be taken into consideration in model selection [71]. It was, therefore, important to evaluate accuracy across predictor size using directly comparable models developed using a GLCM statistic and window size, as illustrated in Fig 5A. A specific comparison between the best model and those that contained fewer bands of entropy GLCM at 5×5 window size showed little difference in accuracy, indicating the potential of using simple models to estimate species diversity. In addition to model simplicity, knowledge of good models that use few predictors (such as those shown in Fig 5A) is significant since these models allow the use of suitable bands and they avoid those that show a high level of uncertainty due, for instance, to atmospheric interference.

Although the effects of the three window sizes (3×3, 5×5 and 7×7) were comparable for entropy GLCM-based analysis, the two smaller window sizes had a marginally better effect on the accuracy (Fig 4). This shows the importance of limiting the size in characterising species diversity at a localised scale such as one considered in this study. Overall, window sizes also did not have a consistent effect on species diversity estimation accuracy across GLCMs used in the study (Fig 3). This observation is also noted by [86] who reported that GLCM statistics have varied output with different window sizes. We illustrated a more focused comparison of the 5×5 window (which was taken as the best) with the 7×7 (Fig 5B) and 3×3 (Fig 5C) window sizes of the entropy GLCM. The better similarity of 5×5 with 7×7 than with 3×3, irrespective of the predictor size, shows the need to use fairly large window sizes to reduce the effect of noise commonly encountered in small windows [87]. It should be noted that the comparison of windows can be conditioned to the GLCM statistics used. For instance, we can deduct from Fig 3 that all predictor sizes of 3×3 window size result in significantly low accuracies than those of 5×5 and 7×7 when dissimilarity GLCM is used, while the three windows yield comparable accuracies for second moment and mean GLCMs.

Finally, the best model (namely, that used entropy, 5×5 and five bands) was compared against the best models of all the seven GLCM statistics. This was done to assess how much these statistics maintain the accuracy of the best model (Fig 5D). The comparisons generally showed low correspondence between the best entropy-based model and those developed from the seven GLCM statistics. However, this should not be construed as weak estimation accuracy for individual GLCM statistics as some of them showed good accuracies (Fig 3). For instance, errors of dissimilarity and variance GLCMs had low correlation with errors of entropy GLCM (*r* = 0.72); however, the three of them were the best predictors as shown in Fig 3. This implies that the models had inconsistent performance on each sample, but their overall accuracies remain similar. This, in turn, suggests the importance of identifying appropriate GLCM for species diversity assessment in a vegetation environment such as the one considered in the current study.

## Conclusions

This study explored the performances of eight GLCM statistical measures derived from reflectance values of WorlView-2 individual bands to estimate species diversity during a dry season in a savanna vegetation type. An exhaustive analysis using the all-possible-subset regression approach showed that entropy GLCM statistic performed better than other statistics in capturing plot level species diversity expressed in the Shannon index scale. This finding agrees with other studies and with expectations since the entropy GLCM statistic exploits the complexity of pixel values within a particular window size. This suggests the preference of entropy in a vegetation environment where there is a good deal of complexity that should not be underestimated. Notably, the yellow band formed part of models that yielded best estimation accuracies in most cases, confirming the importance of this band in discriminating species diversity, particularly in the dry season.

The study further evaluated the accuracies of predictor and GLCM window sizes in estimating species diversity. Predictor size had a remarkable pattern of influence on estimation accuracy. Model accuracy increased until three–five predicting bands were used but stabilised or decreased as more predictors were used per model for most cases of GLCM. Accuracies of models that used fewer predicting variables compared to the best models that used three–five bands were fairly good, indicating the adequacy of limited bands in species diversity estimation. The effect of window size on species diversity estimation varied with GLCM type used in the extraction of representative value. This is due to the fact that the GLCM statistics inherently determine the type and level of similarity or contrast derived from a gray-level neighbourhood of pixels.

Although the findings might not be universal to all images and all vegetation environments, this study provides important observations on the performances of high spatial resolution imagery coupled with GLCM statistics for woody species diversity estimation in dry conditions within a savanna environment. Such an approach should be investigated in different savanna environments as well as other ecosystems that might have more diverse species types. Furthermore, it is worth factoring in external variables (e.g. topographic variables and climate data) in classifying species diversity. It is also important to extend such a study by using higher spatial resolution data than used in the current study. Availability of very resolution data are realized in particular with the development of unmanned aerial systems. In addition, GLCM statistics should also be tested for estimating species diversity at a broader scale using different moderate resolution imagery.

## Supporting information

S1 Data (RAR)
